# Technical and Medical Aspects of Burn Size Assessment and Documentation

**DOI:** 10.3390/medicina57030242

**Published:** 2021-03-05

**Authors:** Michael Giretzlehner, Isabell Ganitzer, Herbert Haller

**Affiliations:** 1 Research Unit for Medical Informatics, RISC Software GmbH, Johannes Kepler University Linz, Upper Austrian Research GmbH, A-4232 Hagenberg, Austria; isabell.ganitzer@risc-software.at; 2 Trauma Hospital Berlin, Trauma Hospital Linz (ret), HLMedConsult, A-4020 Leonding, Austria; herberthaller@gmail.com

**Keywords:** burn size assessment, three-dimensional, estimation accuracy, medical documentation, consequences of inaccurate assessment

## Abstract

In burn medicine, the percentage of the burned body surface area (TBSA-B) to the total body surface area (TBSA) is a crucial parameter to ensure adequate treatment and therapy. Inaccurate estimations of the burn extent can lead to wrong medical decisions resulting in considerable consequences for patients. These include, for instance, over-resuscitation, complications due to fluid aggregation from burn edema, or non-optimal distribution of patients. Due to the frequent inaccurate TBSA-B estimation in practice, objective methods allowing for precise assessments are required. Over time, various methods have been established whose development has been influenced by contemporary technical standards. This article provides an overview of the history of burn size estimation and describes existing methods with a critical view of their benefits and limitations. Traditional methods that are still of great practical relevance were developed from the middle of the 20th century. These include the “Lund Browder Chart”, the “Rule of Nines”, and the “Rule of Palms”. These methods have in common that they assume specific values for different body parts’ surface as a proportion of the TBSA. Due to the missing consideration of differences regarding sex, age, weight, height, and body shape, these methods have practical limitations. Due to intensive medical research, it has been possible to develop three-dimensional computer-based systems that consider patients’ body characteristics and allow a very realistic burn size assessment. To ensure high-quality burn treatment, comprehensive documentation of the treatment process, and wound healing is essential. Although traditional paper-based documentation is still used in practice, it no longer meets modern requirements. Instead, adequate documentation is ensured by electronic documentation systems. An illustrative software already being used worldwide is “BurnCase 3D”. It allows for an accurate burn size assessment and a complete medical documentation.

## 1. Introduction

An accurate assessment of both the burn depth and the burn extent is essential for adequate and successful treatment. In order to determine the burn depth, German-speaking countries differentiate between so-called burn degrees correlating with an increasing depth of a burn of the skin [1,2]:

“First-Degree” burns affect the epidermis and lead to redness and severe pain but do not cause cell death.

“Second-Degree” burns are distinguished between “second-degree superficial” (2a) and “second-degree deep” (2b). Whereas the first type involves damage to the epidermis and the superficial dermis, with blisters, a rosy and recapillarizing wound base, severe pain, and firmly anchored hair, the second type is characterized by injuries to the deep dermis and skin appendages. The wound is comparatively pale and has little or no recapillarization, and the pain receptors are partially destroyed, the pain perception of the patient is reduced, and the hairs are easy to remove.

“Third-Degree” burns lead to complete epidermal and dermal destruction and are accompanied by hair absence and a dry, white, leathery hard wound base without pain.

Historically, a “fourth-degree” burn was also distinguished. In this type of burn, charring occurs, and in addition to the epidermis and dermis, other layers are destroyed, such as the subcutaneous fatty tissue, muscles, tendons, bones, or joints.

In comparison, English-speaking countries classify the burn depth into “superficial”, “superficial partial thickness” burn (equal to second-degree superficial), “deep partial thickness” burn (equal to second-degree deep), and “full thickness” burn (equal to a third-degree or fourth-degree) [2]. Clinical evaluations are never reliable, and “the accuracy of bedside depth assessment is widely considered to be far from optimal” [3] (p. 762).

While in clinical practice, an assessment of burn depth based on these classifications tends to be difficult, a classification based on healing time is usually of higher practical value [3]. Usually, burns healing within one week are categorized as “first-degree” or “superficial” burns; those healing within two weeks are referred to as “second-degree superficial” or “superficial partial thickness” burns. If healing occurs within three weeks or more, the burns are classified as “second-degree deep” or “deep partial thickness” burns, while burns taking even longer to heal but still heal spontaneously from the skin’s appendages are classified in the same group. “Full thickness” burns do not heal from regenerative tissue in the wound but the margins [3]. An exact classification of which burn degree correlates with healing time is not given. It is challenging to differentiate between partial deep or 2b burns and full thickness or third-degree burns.

As described above, in addition to the depth of a burn injury, the burn extent is the second important criterion to be assessed to determine adequate treatment methods. The latter is defined as the percentage of the burned body surface area (TBSA-B) to the total body surface area (TBSA), whereby first-degree burns are excluded. According to different studies, an accurate determination of the extent of a burn injury often proves to be challenging in practice. In most cases, TBSA-B is overestimated [2].

Although both burn depth and burn extent are important criteria in burn medicine, the scope of this paper is limited to the assessment and documentation of burn extent.

## 2. Consequences of Inaccurate TBSA-B Assessment

Inaccurate TBSA-B estimations can lead to considerable consequences. The most important ones are described in the following.

### 2.1. Over-Resuscitation

Underhill [4] first published the suggestion to treat burns with fluids intravenously in 1930. A significant advance in the shock treatment of burn injuries was achieved with feasible fluid resuscitation rules based on the “Parkland formula”, also known as the “Baxter Parkland formula”. Baxter and Shires proposed it in 1968 [5]. In 1979, a conference sponsored by the “National Institutes of Health” (NIH) was concluded with a recommendation to resuscitate burn patients with as little fluid as possible to maintain organ perfusion. Accordingly, the initial fluid therapy in the first 24 h should consist of isotonic crystalloid with a volume between 2 and 4 mL/kg/TBSA-B and should be titrated to ensure the urinary output of 30–50 mL/h [6]. 

Abdominal compartments drew attention because most burn centers used fluid volumes for resuscitation, some of which were significantly above the calculated 4 mL/kg/TBSA-B. Several possible reasons for this are described below.

First of all, there was a trend to optimizing resuscitation to “supranormal values” [7] (p. 416). Small fluid boluses were administered until the cardiac output stopped increasing. According to the motto “the more, the better”, this procedure allowed the application of very high volumes. While the initial findings appeared promising, supranormal values did not yield improved results in a multicenter study [8]. Sympathetic activation by vasoconstrictive substances like catecholamine or angiotensin 2 raises central venous pressure (CVP) and releases arterial natriuretic peptide (ANP). Release appears in the early phase of the burn injury when pain increases blood pressure and triggers tachycardia. ANP mediates the “shredding” of the glycocalyx responsible for hindering vessels from leakage [9,10]. The application of additional fluid can aggravate the “shredding” [11]. Damage to the glycocalyx can cause capillary leakage, which later requires higher amounts of fluid [12]. Low volume and high volume responders could be identified after the first four hours. The difference started after two hours and remained unchanged [13]. Once initiated, “fluid begets more fluid” [12] (p. 234). Urinary control alone fails to hinder severe capillary leakage, as fluid goes into the interstitial tissue, which does not necessarily cause higher blood pressure or higher urine output. Friedrich et al. [14] demonstrated this, examining supra-Baxter resuscitation, and did not observe any difference in 24-h urine output between groups with high and low resuscitation volume. Similarly, Engrav et al. [15] found that there is no rise in average urine output despite increased fluid administration. Regan and Hotwagner [16] recommended the goal of 1 mL/kg per hour for pediatrics below 30 kg and 0.5 mL/kg per hour for those weighing more than 30 kg.

The initial overestimation of TBSA-B enhances all previous effects. Over-resuscitation due to overestimation frequently happens in the course of primary transport, when urine output is not measured, and it often occurs in emergency departments. Overestimation leads to over-resuscitation, and this contributes to capillary damage and edema. The consequences of burn edema can lead to severe complications, which cannot be reversed easily later.

### 2.2. Complications Because of Fluid Aggregation from Burn Edema

While frequently occurring consequences of burn edema, including pulmonary dysfunction and increased intraabdominal and intercompartmental complications, are well described [17], mortality resulting from high-volume resuscitation has not been scientifically proven. However, from abdominal compartments in burns, Strang et al. [18] described an average mortality rate of 74.8%. To the authors’ best knowledge, local complications, such as reduced take rates of dermatomes, have not been investigated by adequate studies up to date, although it is common surgical knowledge [19]. However, it was proved that low volume resuscitation mitigates the probability of multiple organ dysfunction syndromes (MODS) and improves lung function in the early stages [20]. Besides, “suboptimal fluid resuscitation in burn patients leads to greater burn depth and extension of the shock period” [21] (p. 293).

### 2.3. Missing Accuracy in Studies

It is widely accepted that scientific approaches to burn treatment rely on an accurate TBSA-B assessment. Aiming at establishing standards for burn treatment, various international initiatives have been started. Without being exhaustive, these include, for example, the “One Burn One Standard” (OBOS) initiative of the “American Burn Association” (ABA) Committee for the “Organization and Delivery of Burn Care” (ODBC) [22], the “One World, One Burn Rehabilitation Standard” of the “International Society for Burn Injuries” (ISBI) [23], or the standardization initiatives of the German Society for Burn Medicine [1].

The need for standards has been well demonstrated, for example, in the study “Inflammation and the Host Response to Injury” [24]. The development of a standard operating procedure was considered relevant for the study to provide high-quality clinical outcome measures in burn treatment as a basis for further evaluation of genetic and proteomic alterations and their influence on inflammation [25]. As in other studies, the TBSA-B assessment in this study relies on the “Lund Browder Chart” and does not consider inherent errors such as over- and underestimation or inter-rater errors, i.e., two investigators estimating the size of one and the same burn differently. Even in an expert environment where TBSA-B assessments are carried out, the method’s validity and error-based data are essential for further conclusions. As pointed out by Nichter et al. [26] and Wachtel et al. [27], an inter-rater error of 10% can affect the results and significance of studies. Errors like these can easily be avoided and modified using electronic media.

### 2.4. Non-Optimal Distribution of Patients

#### 2.4.1. Distribution to Burn Centers

In deciding whether patients should be transferred to burn centers, the size of a burn injury is the most crucial factor. It is initially evaluated either preclinically or in the emergency room, in surroundings usually not accustomed to the burn treatment. In many cases it deviates significantly from the subsequent TBSA-B evaluation in a burn center [28,29]. Goverman et al. [30] showed that as a result of the initial burn assessment, 59% of pediatric patients of an average age of 4.1 years were given significantly more fluid than according to the later evaluation in the burn center would have been required. Furthermore, this study’s authors reported an overestimation of burn size in 94% of all transferred children, with an average TBSA-B overestimation of 339% by referring hospitals. Reasons for this might be the lacking experience of non-trained medical personnel and the unavailability of special technical equipment [31,32]. Since the “Lund Browder Chart”, which usually results in an overestimation of burn size, was the basis for the assessment, the reported overestimation rate might be higher. However, according to ABA rules, specific percentages of TBSA-B indicate patient transfers to burn centers; hence, an overestimation could result not only in excessive use of resources but also in avoidable costs for transportation [33].

#### 2.4.2. Distribution of Patients in Mass Casualty Situations

In the case of mass casualty situations, the capacity utilization of burn centers is of crucial importance. Therefore, the distribution of burn patients needs to be carefully managed. Inaccurate burn size estimations can lead to non-optimal treatment decisions and complications. Failed distribution to burn centers can hinder treatment for those patients who urgently need it.

## 3. Methods for Burn Size Estimation

**Figure 1:**
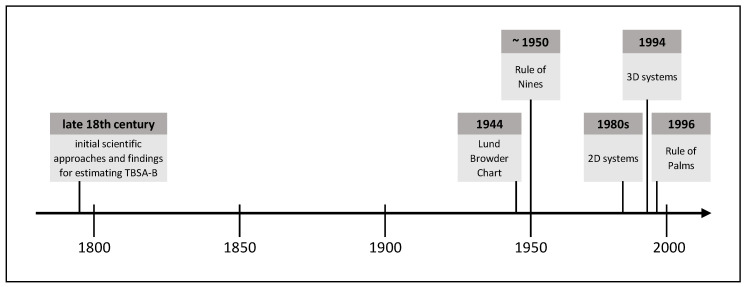
Timeline of methods for TBSA-B determination described in this article.

Due to the frequently occurring inaccurate estimation of the burn size in practice and the associated far-reaching consequences for patients and medical resources, objective methods allowing for a more accurate burn size assessment are required. Over time, various methods have been established, whose development has been influenced by contemporary technical standards. The following is an extract of burn size estimation history and shows methods proven in practice or with high practical potential. Figure 1 shows this extract of TBSA-B determination methods as a timeline.

### 3.1. Initial Scientific Approaches and Findings for Estimating TBSA-B

Documented knowledge about the link between the severity of burns and the survival probability begun in Europe in the late 18th century with Richter’s report in the year 1788. Schjerning gave a rough description of this connection in 1884. The correlation of burn size and mortality was questioned at that time, for example, by Liman [34]. Based on Meeh’s calculations in 1879, Weidenfeld from the University of Vienna established a constant ratio of well-defined body areas to the TBSA. He defined the ratio as proportions and not yet as percentages. Besides, Weidenfeld demonstrated the correlation between burn size and time of early death [34]. Riehl confirmed these findings in 1925 [35].

Without being aware of the work by Weidenfeld, Berkow recalculated the surface area of the body parts of five people with different physiques according to Dubois and Dubois [36]. He observed a mean error rate of 15% in Meeh’s calculations and reported an average error below 5% in his owns. Further, Berkow found that children’s body proportions differ from those of adults and suggested to take this into account. He proposed to name the method of calculating burn size as a percentage of the TBSA according to Weidenfeld and himself, but this was rejected [34].

In 1942, the scientifically based burn treatment became the focus of national interest, not only due to the ongoing World War but also because of the “coconut grove nightclub disaster”. A Burn Research Service was established at Boston City Hospital, and a “National Committee for Burn and Trauma Research” begun its work. TBSA-B determination and the feasibility of a high-quality method became increasingly important. Treating burn shock on the basis of TBSA-B was suggested at the “National Research Council Conference” in 1942 [34].

### 3.2. Lund Browder Chart

Two years later, in 1944, C.C. Lund and N.C. Browder [37] from Harvard Medical School published the so-called “Lund Browder Chart” to improve the calculation of body proportions and to reduce errors. This chart was based on Boyd’s [38] surface area calculations. Lund and Browder defined clear boundaries of body regions and considered different proportions during human growth. Even though many authors have modified the “Lund Browder Chart” [39,40], it has remained in use in its original form until the present.

#### 3.2.1. Description

The “Lund Browder Chart” illustrates the human body’s front and back in a graphic model and assigns different body proportions to different age groups. It shows the boundaries of specific body regions for which different percentages of TBSA are defined. For the calculation procedure, an adapted planimetry is used. Even though the “Lund Browder Chart” does not differentiate depending on sex, weight, height, or body shape, many authors consider it “the most accurate” method [41] (p. 58).

#### 3.2.2. Estimation Accuracy and Criticism

Several authors reported an overestimation of burn extent when using the “Lund Browder Chart”. This could be due to the fact that it is based on just a single physique. Different weight categories and body shapes are not taken into account, nor are changes in body proportions between the respective age groups [41,42].

Additionally, in comparison with 3D methods, burn size assessments using the “Lund Browder Chart” result in severe overestimations [42]. Overestimations tend to be more significant in small burns and smaller in more extensive burns [43]. When using the “Lund Browder Chart”, underestimation of the extent of a burn injury is comparatively rare. It is particularly likely to occur in the case of very extensive burns, as the assessor may tend to estimate the healthy skin areas rather than the burned ones [41].

Furthermore, the use of the “Lund Browder Chart” often leads to high inter-rater errors. Such differences between assessors evaluating the same burn are often related to different environments (e.g., accident and emergency departments, preclinical evaluations, and burn centers), where several errors may occur. In many studies, large differences were found [28,29,44,45], with most of the preclinical evaluations being compared with the evaluation results of the burn centers, which in turn relied on the “Lund Browder Chart”.

The challenge in the evaluation of the “Lund Browder Chart” lies in comparing it with objective measurements. Although not objective either, the “gold standard” for comparisons is typically an experienced senior surgeon at a burn center. Computer-aided planimetry can only improve the percentage of burns in identified areas without calculating the actual extent since the projection error is inherent in this method. However, computerized techniques, such as 3D measurements and stereogrammetry, can be appropriate. Another way to evaluate burns is to use paper squares [41]. Klippel [46] expressed doubts regarding the validity of the “Lund Browder Chart” since it has not been validated and is based on rather old data.

### 3.3. Rule of Nines

Continuing with the history of burn size estimation, another method still used in practice is the so-called “Rule of Nines”, originating from a discussion between friends Wallace and Pulaski in 1949. At a symposium of the “National Burns Research Council” in Washington in 1950, Pulaski presented a slide of the “Rule of Nines” based on a collaboration with Tennison, leading to the fact that most American authors consider these two to be the original authors of the rule [34].

#### 3.3.1. Description

In his publication in 1951, Wallace [47] assumed for different body parts the following proportions of the TBSA: arms 9% of the TBSA each, legs 18% each, chest and back 18% each, head and face 9%, neck 1%, and genital area 1%. Like the “Lund Browder Chart”, the “Rule of Nines” ignores differences in sex, weight, height, and body shapes.

#### 3.3.2. Estimation Accuracy and Criticism

Initially, the “Rule of Nines” was meant for preclinical application in the event of disasters and mass casualties. When using this rule, the burn extent is overestimated in many cases [27,48]. For example, Giretzlehner et al. [49] reported a mean overestimation rate of 138% using the “Rule of Nines” and the “Lund Browder Chart”. Furthermore, overestimation mostly occurs in patients with an increased body mass index (BMI). For patients weighing more than 80 kg, it is more promising to apply a “Rule of Fives”, and below 10 kg to apply a “Rule of Eights” [50]. Disregarding different body shapes usually results in an overestimation of extremity burns and underestimating upper body burns [51]. In comparison to the results of 3D scans, the back and the torso of normal-weighted patients are overrepresented by the “Rule of Nines” [52]. Additionally, a high inter-rater error can be expected [42].

### 3.4. Rule of Palms

The “Rule of Palms” by Rossiter et al. [53] relies on the original “Lund Browder Chart”. It can be applied either alone or in combination with other methods such as the “Lund Browder Chart” to estimate the TBSA-B of a specific body region.

#### 3.4.1. Description

In its simplest form, the “Rule of Palms” states that the patient’s hand’s surface accounts for approximately 1% of the TBSA. Due to different understandings on whether the palm should be calculated including or excluding fingers, this rule is used inconsistently in practice [53]. It is mainly applied to measure the size of reasonably small burn injuries [34].

#### 3.4.2. Estimation Accuracy and Criticism

Usually, the “Rule of Palms” results in an overestimation of the actual burn extent. Fundamental differences are depending on sex and age. Assuming normal BMI, the palm of a man represents an average of 0.81% and the palm of a woman 0.67% [53] of the TBSA. The isolated palm without fingers amounts to 0.52% for males and 0.43% for females [53]. In children aged between one and 13 years, the palm with fingers accounts for 0.92% and the palm without fingers for 0.52% on average [54].

The BMI influences the “Rule of Palms” since the palm’s actual area does not change to the same extent as the TBSA with a BMI above 30 [55] in neither men nor women. Butz et al. [56] described the percentage of the palmar surface area of the TBSA depending on the BMI. In average weight persons with a BMI from 18.5 to 24.9, values between 0.87% and 0.91% in women and between 0.95% and 0.99% in men were reported. In persons with a BMI of 40 and higher, the values ranged between 0.67% and 0.70% for females and between 0.68% and 0.72% for males.

In the practical application of the “Rule of Palms”, the degree of overestimation varies. For example, Hintermüller [31] found an average overestimation of 70.88% of the actual area, with seven wounds overestimated in the range from 41.55% to 173.08%. The “Rule of Palms” could be a reason for the substantial overestimation of up to 100% in the emergency departments as it was described by Laing et al. [57]. An explicit dependency on the specialization and grade of assessors in emergency departments was described. Estimations were between 7% and 133% too high, even though applying a “Lund Browder Chart”, possibly due to the “Rule of Palms” to assist in “Lund Browder Chart” evaluation. Besides, Cone [58] described a mean overestimation rate of 75% when referring physicians. When combining the “Chinese Rule of Nines” with the “Rule of Palms”, Sheng et al. [59] reported an overestimation in the range from 12% to 30% in 17 wounds evaluated by four surgeons.

Summing up, a palm does not result in exactly 1% of the TBSA. Not least because of the inaccurate definition and different usage in practice, a high overestimation rate can be expected when applying the “Rule of Palms”. Furthermore, a low inter-rater reliability can be expected [42].

### 3.5. Two-Dimensional Computer-Aided Systems

In the course of technical advances, the development and implementation of IT-based systems started at the beginning of the 1980s. Wachtel et al. [60] and Nichter et al. [26] were among the first to employ electronic systems.

#### 3.5.1. Description

Two-dimensional (2D) computer models rely on simple human body drawings on a computer screen. These models do not consider the human body’s three-dimensionality, and in many applications, it is not possible to capture the lateral and other parts of the body. Moreover, 2D models usually do not depict individual differences in sex, weight, height, and body shape. Nevertheless, they are easy to handle but can only provide a rather rough overview of the burn type and the affected areas, particularly on the lateral parts of the body. In many cases, they miss the actual extent of the burned area.

The planimetry type can be distinguished between simple planimetry and adapted planimetry. While the former is related to a simple pixel count in a 2D image and is used in some electronic devices, the latter is a pixel count in a 2D image that is additionally corrected by a particular percentage recommended for a specific body region.

#### 3.5.2. Sample Applications

An example of a 2D system is the smartphone application “Mersey Burns”. The app was approved as a medical device by the “U.K. Medicines and Healthcare Regulatory Agency” [61,62], although the TBSA-B estimation error was not examined. The basis for the electronic calculations is a two-dimensional “Lund Browder Chart” [62].

Besides, “SAGE II” (“Surface Area Graphic Evaluation II”) was developed by Parshley in 1987 and relies on an adapted planimetry of “Lund Browder”. It uses 2D charts that can be adapted to age, weight, and height [63]. It is available online for free single evaluations or as a licensed version for multiple observations. Unfortunately, the web application is no longer running in modern web browsers. Other examples for adapted planimetry include the “Rule of Nines” [64], the “Rule of Fives” [51], the “Lund Browder Chart” [37], and related charts, and some computerized charts partly accommodated to the individual characteristics of the patient. Furthermore, most apps for smartphones rely on 2D calculations that are corrected by the percentages of “Lund Browder”.

#### 3.5.3. Quality of Estimation Reliability

**Figure 2:**
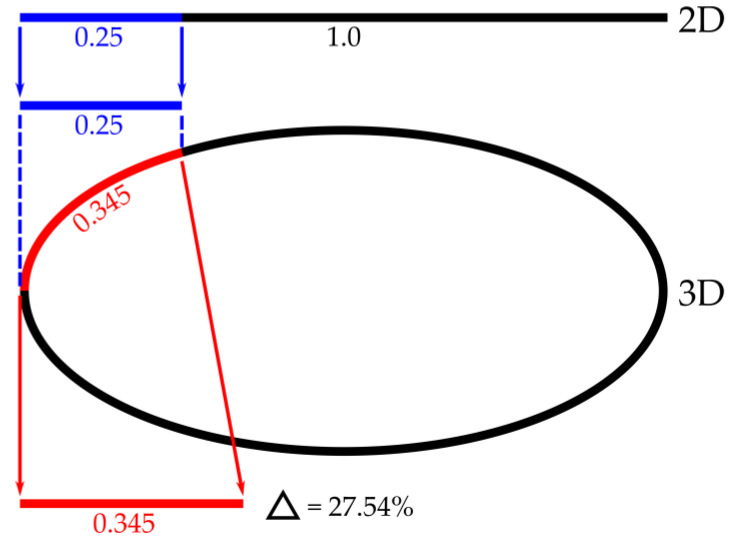
Comparison of 2D and 3D calculation of burned area.

In terms of estimation accuracy, 2D systems have the disadvantage that projection errors occur. When displaying a lateral burn, e.g., a burn in the lateral abdominal area, a 2D system would display the body in an anterior and posterior half. This results in a distortion and thus an incorrect calculation of the extent of the burn. Figure 2 shows an example where a quarter of the anterior trunk is affected. In the 2D perspective this would only be 0.25 of the body width. Using a 3D system, the same represents the arc length of 0.345, which is Δ = 27.54% larger (Δ represents the difference in percentage).

### 3.6. Three-Dimensional Computer-Aided Systems

In 1994, Lee et al. were the first to develop and describe 3D (three-dimensional) systems [65,66]. The significant advantages of 3D models are the lateral areas’ presence and the possibility of adapting to the patients’ characteristics and better representing the real patient. Three-dimensional systems prevent the methodological error of reduction to a 2D drawing. Typically, 3D systems allow for an adaptation to sex, weight, height, and body shape. Available systems achieve a high validity in adults with a BMI below 30, but there are some limitations with very obese burn patients and unusual body proportions. The more accurate the model is, the more precise the adaptation to the individual body shape and body characteristics of the patient [67].

Systems based on individual measurements should provide an exact picture of the patient’s body to be assessed, considering the individual body characteristics. A precise 3D scan appears to be a complete individual and accurate 3D model. However, due to the time-consuming process, they were mainly applied in studies, having deficiencies, and showing other methods’ weaknesses [68].

Model-based systems use models that are modified to sex, height, weight, age, and body shape. Based on the measurements, the model is selected from a library either by the user or partially or fully computer-aided. As far as the computer system allows, the model is then adapted to the individual characteristics.

#### 3.6.1. Three-Dimensional Images

3D images, together with a size objectification (e.g., a ruler, a fixed distance between photos, or a grid pattern [69]) have a high potential to accurately assess the extent of burns, particularly of small ones. Since the values are expressed in square centimeters, calculating the TBSA-B percentage requires a TBSA estimation formula. Therefore, this method is only suitable for small burn injuries [70].

#### 3.6.2. Three-Dimensional Scans

Three-dimensional scans provide a good support to accurately determine TBSA-B since once calibrated, these systems indicate an area’s absolute size.

Partial scans: One field of application of partial scans is the production of compression devices, e.g., for the head. The 3D scan of the face and scars replaces the traditional plaster cast method. 3D printers can produce compression devices. Unique algorithms must determine the degree of compression.

Total scans: In the case of burn injuries or unusual body proportions, the 3D scan requires a full body scan of each aspect. Since a scan is only able to determine the body surface, scans of the armpit, perineum, and others are also required. Thus, an average of eight scans is needed to precisely reconstruct a body [71]. The individual scans have to be combined into one whole-body scan image that requires evaluating the composition procedure. Three-dimensional scans from point clouds need to be stored in a model to evaluate changes on the surface. All surface points can be tracked over the timeline.

So far, no useful 3D scan has been published for whole-body scans in the context of burn injuries. “BurnCalc” shows the accuracy of 3D scans but does not indicate their application in the case of patients under ventilation and sedation. The storage in a point cloud does not track a specific spot of the surface over different scans [59].

#### 3.6.3. Sample Applications

“3D Burn Vision” is a software (V1.0, University of Chicago, Chicago, CA, USA) developed at the University of Chicago and was sponsored by the EPRI (“Electric Power Research Institute”, Washington, DC, USA). It offered many advanced functionalities, such as adapting to the individual body characteristics of the patient. It allowed for zoom and rotation, had a morph function, joints could be moved, the results could be stored in an electronic database and it enabled multiple observations [63]. “3D Burn Vision” allowed for a documentation of burn degrees and the area of allografts and autografts. Several use concepts could not be realized due to funding [42].

The research project “BurnCase 3D” allows for 3D registration and documentation of burn patients. It was initiated as a student project in the year 2001 and is currently operated by RISC Software GmbH (V2.6, RISC Software GmbH, Hagenberg, Austria) that is 80% a subsidiary of the Johannes Kepler University in Linz and 20% owned by Upper Austrian Research. For an annual fee, members can use the full functionality, influence the ongoing project and receive support. The software and database run on modern Windows versions and can be used as a standalone version or as a server with multiple clients serving different departments or even hospital networks.

The development of “BurnCase 3D” was supported by numerous medical partners. It is the basis for implementing a new software framework for application-oriented research in the documentation and treatment of burns. This framework is being developed within the ongoing international follow-up research project “SenseBurn” (“EUREKA-2 Eurostars”). The latter is characterized by numerous qualities that allow for easy and accurate estimation and burn injuries documentation:

Platform independence: The development resulted in software that runs on Android, iOS, Windows, Linux, MacOS, and directly in a web browser without requiring installation.

Patient-Specific 3D models: The creation and adaptation of patient-specific 3D models for different body shapes and proportions were realized with hand-crafted expansion vectors deforming a 3D model from a uniform model collection. These unified 3D models allow for continuous documentation, even if body proportions require a different base model. Personalized models considerably increase documentation quality.

Pose adaptation: The implementation of an automated pose adaptation of the model was realized to facilitate efficient clinical routine use. For this purpose, the joints’ location and position are extracted from a single RGB image using machine learning algorithms. Due to the exact match of pose and shape of the 3D model, the wound surfaces’ transfer is possible with minimal effort and high accuracy.

Exact transmission of wound areas without artifacts: With the development of a new method for wound annotation independent of the mesh resolution of the 3D model, it is now possible for the first time to document tiny areas or slight changes over time.

New possibilities: The implementation of new algorithms such as machine learning provides the opportunity to use future technologies.

Nichter et al. already implemented graphics tablets for drawing burns in 1984 [26]. Technological advances decreased the size of computers and monitors, providing more power than the large ones of earlier times. As a result, many applications were created to perform TBSA calculations based on the principles described later [32,62]. More recently, platform-independent TBSA calculators have been developed as apps for smartphones and tablets.

Another system is “BurnCalc” that was developed by Sheng et al. [59]. It allows for 3D scanning, 3D reconstruction, and interactive TBSA-B calculation. It is a high-tech approach demonstrating the high accuracy of 3D systems. However, its feasibility has not been proven in clinical application.

The programs and apps described above can guide burn treatment, but their implementation is limited because of medical device regulations and the lack of certifications as a medical product by the Food and Drug Administration (FDA) or/and the European Community.

#### 3.6.4. Quality of Estimation Reliability

Different results of calculators can be explained by different methods used. Usually, 3D systems show a little inter-rater error and a high intraclass correlation.

Hintermüller [31] compared an electronic 2D system (“Mersey Burns“) with an electronic 3D system (“BurnCase 3D“). As the results showed, “Mersey Burns” was associated with a mean overestimation rate of 32.16% to the ground truth. “BurnCase 3D” showed an overall difference to the ground truth of −4.53%. Thus, in terms of accuracy, the 3D system outperformed the 2D method. Goldberg et al. [72] reported similar results.

Parvizi et al. [67] demonstrated the high reliability and validity of “BurnCase 3D”. In their validation study, artificial burn wounds of known size were applied to different ages and sex models. The study showed an average overestimation rate of burn extent of 0.4% in the pediatric model, 2.8% in the female model, and 1.5% in the male model.

Hintermüller [31] concluded that IT systems could help minimize potential errors or deviations from the actual burn extent, mostly when the less experienced medical staff performs the assessment.

As already pointed out, overestimation of the burn extent often leads to over-resuscitation, which in turn is a frequent cause of complications such as burn edema or capillary damage. Since 3D systems allow a very accurate calculation of the burn extent, their application can reduce such complications.

Adaptations of conventional methods such as the “Rule of Nines” or the “Lund Browder Chart” were developed to meet the challenge of varying body proportions during growth. Nevertheless, the developments led to inaccurate results in measuring TBSA-B, often with overestimation of burn extent or low inter-rater reliability [73]. 

Haller et al. [42] scored the extent to which different TBSA-B assessment methods meet future requirements in burn care. Traditional manual methods have the advantage that they are easy to use and require little equipment. However, compared to electronic 3D systems, they do not consider differences in individual physiology and are therefore less accurate.

Computer-based systems can still be prone to a methodological error, which occurs when transferring a 3D surface to a 2D model. The percentage assigned for a specific area by “Lund Browder” is not accurate for an individual. Nevertheless, inter-rater error is lower than manual and brain work, both in 2D and 3D systems [31,60,67,74].

## 4. Documentation

### 4.1. Medical Documentation

Any documentation aims to make the documented facts available. In most cases, such documentations focus on gathering data itself rather than making existing data available.

Documentation requirements are far beyond traditional paper-based documentation, even if converted into computer-based forms. Experts have well defined the required medical features for wound documentation. Even if there is plenty of literature dealing with chronic wounds [75,76], we found no validated standards for burn wound documentation of all necessary aspects.

From a medical point of view, the requirements to ensure modern and up-to-date wound documentation should be adapted to burn wounds and at least consist of:

- Medical history and general status of the patient with all its features;
- Recent and frequent photographic documentation to evaluate changes in the wound;
- Wound assessment with all its features;
- Course of healing;
- Documentation of therapeutic measures and their efficacy;
- Results of follow-ups;
- Traceability and verification of authors.

### 4.2. Standards Required for Data Analysis

Due to a possibly low number of cases, it is beneficial to analyze multiple burn centers’ collected data. A shared merged datastore is necessary considering data security concerns. Merging data from multiple sources is a significant challenge because institutions use different software tools, different types of data storage structures, different conventions, unequal periods, and different levels of data aggregation. Commonly accepted data standards and compliant implementation of all parties’ systems are necessary [77]. Solutions to get standardized data of different institutions might be the usage of a standardized documentation system like “BurnCase 3D” or the transforming of each single data collection to a unique one.

### 4.3. Existing Documentation Systems

Although paper-based documentation has significant shortcomings compared to electronic wound documentation [78], many institutions still use paper forms (or free-text electronic forms). The literature shows that most clinical systems do not meet the known requirements for successful burn documentation.

Many existing documentation systems use predefined terms without indicating their sources or have deficiencies in capturing a patient’s complete medical history due to the lack of standards. Besides, most of them do not include the ability to perform statistical analysis of the collected data simultaneously, and only a few systems can collect data via mobile devices [79].

#### 4.3.1. Electronic Documentation

Paper-based wound documentation is no valid alternative. Up-to-date wound documentation brings up more challenges and requirements.

Electronic documentation systems proved qualitative and quantitative advantages in several studies. They enhance documentation quality, reduce documentation errors, and result in positive attitudes among medical staff. Advantages like better availability and evaluability of the collected data, the more direct exchange of information (for consultation of experts), easier access to resources, and creation of new medical knowledge were described by Törnvall et al. [78] and Kinnunen et al. [75].

Electronic documentation does not necessarily generate scientifically useful data. Even though free-text documentation has flexible terms, dynamic expressions, and more effective recording through dictation, it also has serious shortcomings. Due to linguistic diversity and the lack of structure, there is no possibility to check the quality and completeness of the documentation. Additionally, free-text documentation often assumes besides text other implicit information, and data analysis beyond single patients is complicated and error-prone [80].

Thus, to ensure an optimal basis for data evaluation, a documentation system should provide structured data for selection and avoid free-text documentation. Structured recording allows for an exact recording of facts of defined scopes, such as the complete patient, an exceptional condition, or a single examination. Although the information given might sometimes be less comprehensive than in free-text documentation [80], the quality of structured data is superior due to uniform terminology.

Specifically, for the documentation of burn injuries, a relatively comprehensive documentation system has been developed. “BurnCase 3D” provides a library of 3D models, which can be adapted to sex, age, height, and weight. This system replaces estimation by automatically calculating the burned surface area (TBSA-B) regardless of body shape. The 3D model can be moved, rotated, and scaled. Users can transfer burn wounds from superimposed photos to the 3D model. “BurnCase 3D” enables full documentation of the entire treatment process from initial assessment to the outcome. Parvizi et al. [67] proved “BurnCase 3D” as a valid and reliable tool for TBSA-B determination and documentation. 

From the perspective of medical care management, a significant advantage of 3D systems such as “Burncase 3D” is the accurate documentation of the wound healing process over time, so that the course of treatment can be monitored and appropriate follow-up treatment measures can be taken.

For significant improvements in modern burn care, it is essential to optimize TBSA-B methods and have complete comparable documentation sets.

#### 4.3.2. Mobile Documentation

Mobile devices change the paradigm of data acquisition. Smartphones and tablets facilitate the remote exchange of medical information to assist diagnosis and treatment [81]. These devices are operated on different software platforms (e.g., Android, iOS, and Windows). A modern, state-of-the-art computer documentation system must be able to handle different operating systems. A platform-independent (e.g., HTML5-based) solution is required for this reason.

#### 4.3.3. Photo Documentation

Recent and frequent photographic documentation is necessary to evaluate changes in the wound. Changing staff requires up to date information to avoid unnecessary pain and disturbing dressing changes. For scientific evaluation of the wound, photographic documentation is essential. Infrared and multispectral imaging are examples for particular photographic applications.

Assignment of photos to an actual body localization must be intuitive to be beneficial. High-quality photographs stored in a PACS (picture archiving and communication system) with an automated assignment to all available data (e.g., patient body region and burn condition, date) is necessary to be useful in medical routine. Burn documentation must be able to interact with PACS in the most automated way. 

## 5. Conclusions

The article shows up the consequences of inaccurate TBSA-B assessment, which underlines the importance of appropriate methods. During the last century, several approaches have been developed to meet the requirements and enhance the burn size determination.

Several traditional methods like the “Lund Browder Chart”, the “Rule of Nines”, or the “Rule of Palms” are still of high practical relevance due to their simplicity and availability. Literature has shown some limitations of those methods; therefore, it is important to be aware of them.

In accordance with the technical achievements, modern computer-aided methods have proven to be superior to conventional methods. Computer-aided assessment and documentation systems have the potential to enable the way to a holistic structured and standardized documentation, which is essential to create new medical evidence, which brings the burn treatment a step forward.

To enable an even more comprehensive determination and documentation of burn injuries, 3D systems could be combined with various methods for determining the burn depth in the future. Examples of the latter are already existing methods such as laser Doppler or multispectral analysis. In the authors’ view, such combinations could enable a wide range of new possibilities in burn medicine.

## Abbreviations

2D	two-dimensional
3D	three-dimensional
ABA	American Burn Association
ANP	arterial natriuretic peptide
BMI	body mass index
CVP	central venous pressure
EPRI	Electric Power Research Institute
FDA	Food and Drug Administration
ISBI	International Society for Burn Injuries
IT	information technology
MODS	multiple organ dysfunction syndromes
NIH	National Institutes of Health (United States)
OBOS	One Burn One Standard
ODBC	Committee for the Organization and Delivery of Burn Care
PACS	picture archiving and communication system
RISC	Research Institute for Symbolic Computation
SAGE II	Surface Area Graphic Evaluation II
TBSA	total body surface area
TBSA-B	percentage of the total body surface area burned
